# Cor Triatriatum Dexter Associated with an Ostium Primum Atrial Defect and Left-Sided Opening of the Coronary Sinus in a Stillborn Fetus

**DOI:** 10.3390/jcdd10090370

**Published:** 2023-08-28

**Authors:** Silvia Farkašová Iannaccone, David Sedmera, Alžbeta Ginelliová, Peter Bohuš, Lucia Mistríková, Daniel Farkaš

**Affiliations:** 1 Department of Forensic Medicine, Faculty of Medicine, Pavol Jozef Šafárik University, 041 80 Košice, Slovakia; silvia.iannaccone@gmail.com; 2 Institute of Anatomy, First Faculty of Medicine, Charles University, 128 00 Prague, Czech Republic; 3 Medico-Legal and Pathological-Anatomical Department of Health Care Surveillance Authority, 043 74 Košice, Slovakia; e.ginelli@gmail.com (A.G.); farkas.dany@gmail.com (D.F.); 4 Department of Pathology, Louis Pasteur University Hospital, 040 01 Košice, Slovakia; peterbohus9@gmail.com; 5 Department of Heart Surgery, East Slovak Institute of Cardiovascular Disease, 040 11 Košice, Slovakia; lucia.mistrik@gmail.com

**Keywords:** cor triatriatum dexter, ostium primum atrioventricular septum defect, left-sided opening of the coronary sinus, coronary artery bridging

## Abstract

Cor triatriatum is a very rare cardiac malformation characterized by the presence of an abnormal interatrial membrane separating either the left or right atrial chamber into two compartments. It can be associated with other cardiac defects and is often symptomatic in childhood. The signs depend on the size and position of the interatrial membrane and other associated malformations. Here we report a case of right-sided cor triatriatum associated with an ostium primum-type interatrial septum defect and left-sided opening of the coronary sinus in a fetus. The cause of intrauterine death was asphyxia due to total placental abruption.

## 1. Introduction

A heart with three atria (cor triatriatum, CT) is a rare cardiac defect that can affect either the left atrium (cor triatriatum sinister, CTS), or, even more rarely, the right atrium (cor triatriatum dexter, CTD) [1]. CT is characterized by the presence of an abnormal intraatrial membrane, which subdivides the chamber into two compartments [2] and is a result of abnormal embryonic development [1]. This defect may, or may not, present with clinical symptoms [1]. In this case report we describe a highly unusual case of CTD associated with coronary sinus opening into the left atrium in a stillborn fetus in the 28th week of gestation, where the cause of death was complete placental abruption.

## 2. Case Description

### 2.1. Maternal Characteristics

The proband was a 30-year-old woman in her fourth pregnancy. The children from her previous pregnancies (aged 8, 7 (twins), and 5 years) are alive and physically healthy. One was diagnosed with autism spectrum disorder. The patient was admitted to hospital in the 28th week of gestation for sudden-onset abdominal pain and loss of fetal movement. The ensuing gynecological examination found intrauterine death due to complete placental abruption.

### 2.2. Fetal Characteristics

The delivered fetus was of female sex (1310 g, 38 cm) without any overt external malformations. An autopsy was performed after 5 days. External examination found slight autolysis. Abdominal, as well as thoracic, organs were correctly localized and of normal situs and configuration. Due to the collapse of the veins, any obvious anomalies of the venous system could not be determined with certainty.

### 2.3. Macroscopic Description of the Heart

**Figure 1:**
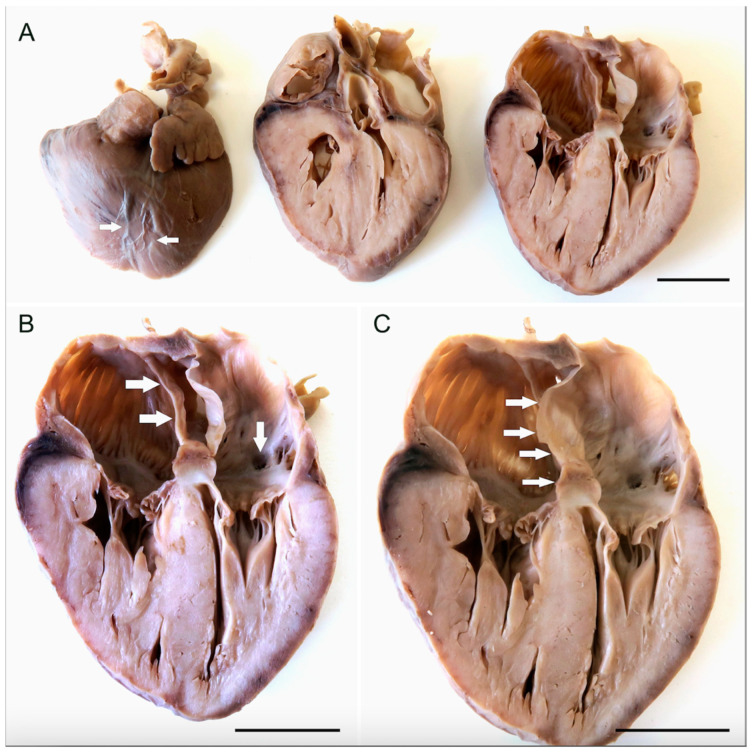
Slices of the fetal heart in anterior view. (**A**) Arrows indicate the bridging of the coronary arteries on the anterior heart surface. (**B**) The last slice of the series, with horizontal arrows pointing to the intraatrial membrane and the vertical one to the anomalous opening of the coronary sinus into the left atrium. (**C**) Enlargement of the most posterior slice of the heart viewed from the front. Arrows indicate the ostium primum type ASD in the interatrial septum. Scale bars—1 cm.

**Figure 2:**
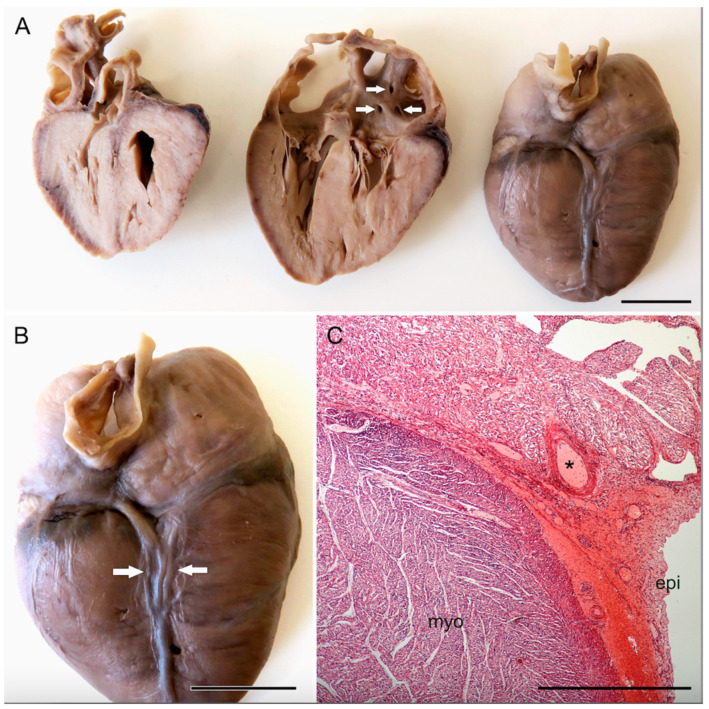
Posterior views of the heart slices. (**A**) Arrows in the middle slice indicate multiple and high openings of the anterior cardiac veins into the right atrium. (**B**) Enlargement of the posteroinferior surface of the heart showing the duplication (arrows) of the middle cardiac vein, entering the coronary sinus that opens anomalously into the left atrium, and the small cardiac vein. (**C**) Histological section (H&E staining) of the posterior part of the heart, showing the subepicardial sinusoids in the posterior interventricular groove and signs of recent bleeding around them. The asterisk marks the right coronary artery in the atrioventricular groove. Scale bars—1 cm (**A**,**B**) and 1 mm (**C**).

The heart dimensions were 3.5 cm × 3.0 cm × 1.3 cm, and the weight was 8.6 g. There were fresh blood effusions around the coronary arteries. The anatomical arrangement of the thoracic aorta, its course and branches including the arterial duct was normal, as was the position of the pulmonary trunk, the caval veins, and the pulmonary veins. Myocardial bridging of the coronary arteries was detected on the anterior wall of both ventricles (Figure 1). There was an abnormal position of the middle cardiac vein on the posteroinferior heart surface, which formed a plexus coalescing into two main branches: one emptying into the coronary sinus, and the other into the small cardiac vein (Figure 2). The coronary sinus did not reach all the way to the posterior wall of the right atrium. The heart was perfused by formaldehyde several times and immersed in the same fixative to facilitate further detailed examination. Due to its dimensions, only two sections were performed on the heart. Opening of the right-sided structures revealed a large atrial septal defect of the ostium primum type (Figure 1C), located anteriorly. To the right of the interatrial septum and running parallel with it, an intact, unfenestrated delicate intraatrial membrane was found, separating the right atrium into two compartments of unequal size—a larger anterolateral part, and a smaller posteromedial one (Figure 1B). There was a communication between these two compartments anteriorly. The openings of the superior and inferior caval vein were located between the right-sided intraatrial membrane and the interatrial septum. The membrane thus almost separated those openings from the tricuspid valve orifice and the entrance to the right auricle. The entrance to the right auricle was located opposite the anterolateral part of the right atrium. The opening of the veins draining the right ventricle and atrium was located anteriorly on the endocardial surface between the superior caval vein and the right auricle (Figure 2A). A foramen with a 0.35 mm diameter, continuous with the cardiac veins, was located above the posterior leaflet of the mitral valve. It was, by definition, termed the coronary sinus (Figure 1B). A distinct left-sided atrial myocardial ridge (part of the pectinate muscles network) was present above the orifice of the coronary sinus. There were several outpouchings reaching to the epicardium in an area of 0.4 × 0.3 cm between the coronary sinus opening and the edge of the mitral fibrous annulus. Together, they formed a complex subepicardial network of sinusoids (Figure 2C). However, the shape and morphology of the mitral valve were entirely normal. The thickness of the right ventricular free wall ranged between 3 and 4 mm, that of the interventricular septum was 5 mm, and the left ventricular free wall was 3 to 4 mm thick.

### 2.4. Microscopic and Laboratory Examination

A histopathological examination of the internal organs revealed their immaturity; there were signs of recent bleeding into the epicardium (Figure 2C), lungs and kidneys, and signs of extramedullary hematopoiesis. A toxicological examination of the fetal blood showed no traces of ethanol or other toxicologically significant substances. Complete placental abruption was diagnosed as the cause of fetal demise.

### 2.5. Summary of Findings

Together, these findings constitute a rare case of cor triatriatum dextrum associated with anteriorly located ASD of septum primum type and left-sided opening of the coronary sinus. In addition, anomalous myocardial bridges over the coronary arteries and the opening of the right ventricular veins anteriorly into the right atrium and sinusoids around the coronary sinus opening present malformations of the heart vasculature.

## 3. Discussion

The less frequently used course of fetal heart dissection after formaldehyde fixation using two vertical cuts has some inherent advantages as well as disadvantages. We used the approach described above since we are trying, in collaboration with clinicians, to ascertain to what degree it is possible to correlate clinical and pathological findings, including intravital and postmortem heart dimensions. This includes the results from prenatal ultrasound investigations. Such intravitally obtained changes in fetal cardiovascular systems could then guide the autopsy to focus on less common or hidden cardiac defects, cardiomyopathies or vascular anomalies. In select cases, it is possible to follow the dynamics of pathogenesis of the defects or specify the timing of the pathological changes that lacked a clear morphological correlate intravitally. In this particular case, however, no such prior information was available, and the autopsy findings were purely accidental.

The classification of CTS is based upon the course and location of the intraatrial membrane using the system of Loeffler or Lama, resulting in three categories of CTS [3]. There are, in most cases, other associated cardiac defects such as ostium secundum type ASD and a VSD [2], anomalous pulmonary venous return [4], and very rarely a division of the right ventricle [5]. In the present case, CTD was associated with ostium primum ASD. In the autopsy series, CTD represents 0.4% of total CHD [6] and less than 0.1% of clinically diagnosed CHD [7]. In our institution, no such case had been diagnosed in the past 20 years (over 30,000 autopsies, both pediatric and adult). Failure of the right venous valve regression is considered to be the etiopathogenetic mechanism for the generation of CTD. This results in an anterolateral and posteromedial subdivision of the right atrium. A diagnosis of CTD can be made at any age, often accidentally [3]. We have not found, however, any reports of an accidental diagnosis of CTD during a fetal autopsy. Clinical signs of CT depend on the size of the membranes, their exact position and any fenestrations therein. The symptoms are due to the level of obstruction of the vessels opening into the right atrium by the membrane and the presence or absence of ASD [8]. CTD could thus manifest as an obstruction of the right atrial inflow [3] or cyanosis [8,9,10]. Echocardiography complemented by a CT scan is used in diagnosis, but it could be still missed [2,5]. Cardiac catheterization with angiography is also used as a complementary method [11], but even a combination of these modalities may not suffice to confirm the diagnosis [12]. Treatment is dependent mostly on the associated defects and is performed mostly through a surgical [4,13] or percutaneous [14,15] disruption of the membrane.

Some authors describe a higher occurrence of CTS in boys, and in one case a higher incidence of CTD was reported in girls [2], but also in boys [10]. CTD was in some cases associated with a partial anomalous pulmonary venous return, and ASD of ostium secundum and sinus venosus type [2]; in our case, however, the ASD type was that of ostium primum. The coronary sinus is in most cases a relatively constant vein starting by the union of the great cardiac vein and left oblique atrial vein (former left superior caval vein) that empties to the right atrium [16]. Detailed knowledge of its anatomy is of particular interest in patients undergoing various cardiac interventions [17]. Coronary sinus anomalies are infrequent and include its absence [18,19], enlargement or hypoplasia [20], unroofed coronary sinus [20,21,22] and its abnormal drainage to the left atrium [23,24,25,26], as we observed in the present case. The opening of the coronary sinus into the left atrium can manifest by a varicose dilation of the cardiac veins due to increased left atrial pressure propagating to the cardiac venous network [24]. Venous drainage of the right ventricle is into the anterior wall of the right atrium in the form of prominent anterior cardiac veins, which is an infrequent finding in the autopsy and can less frequently be clinically diagnosed by a CT scan [27].

Concerning the etiology of abnormal cardiac morphogenesis, it is difficult to speculate in the case of CT due to its rarity. In general, more than 50% of all CHD cases still fall into the unknown category [28]. Most cases with known etiology are believed to be caused by genomic abnormalities, including mutations in genes important for heart development such as transcription factors [29] or structural heart genes [30], but also maternal environmental factors such as diabetes [31], a high-fat diet [32], ethanol abuse [33] or exposure to other teratogens including prenatal viral infections (recently reviewed in [28]). The defects of atrial septation, as noted in our case, are far more common in mammals than in other vertebrates, probably due to its higher complexity with primary and secondary septa [34].

## 4. Conclusions

In this case report we described a rarely diagnosed picture of cor triatriatum dextrum associated with an ostium primum type atrial septal defect and left-sided opening of the coronary sinus. Such a combination had not yet been described in the literature, and we believe that a special dissection protocol including formaldehyde fixation was instrumental in this diagnosis. Our findings point out that even in combination with previous fetal clinical imaging, the diagnosis is not obvious, so it is advisable to keep this possibility in mind in the setting of unclear clinical symptoms.

Since our case was an accidental finding with no prenatal ultrasound data to compare with, it is hard to make any recommendations for clinical management. Obviously, it is desirable to have as high a percentage of expectant mothers screened by ultrasound as possible, with all suspicious cases referred to a specialized center for a more detailed examination. We also must not forget the importance of the pathological examination of stillborn fetuses, as it may together with clinical data provide correlates for diagnoses not encountered in the postnatal population.

## Data Availability

Primary data available from the corresponding author upon reasonable request.
